# The Nickel Age in Electrocatalytic Anodic Reactions

**DOI:** 10.1021/jacsau.6c00252

**Published:** 2026-06-30

**Authors:** Toufik Ansari, Arindam Indra

**Affiliations:** Department of Chemistry, 79203 IIT (BHU), Varanasi UP-221005, India

**Keywords:** Ni-based electrocatalysts, structural evolution redox behavior, anodic reactions, in situ and ex situ

## Abstract

In the last three decades, the superior performance of Ni-based electrocatalysts over other transition-metal (TM)-derived materials has been established for different anodic reactions, such as the oxygen evolution reaction (OER), organic oxidation, biomass valorization, plastic reforming, small molecule activation, etc. In the alkaline electrolyte, Ni-based catalysts are transformed into different active crystal phases of Ni­(O)­OH (β, γ, or their mixture) with a wide variation in the local geometry of Ni (octahedral, square pyramidal, square planar, etc.), coordination environment, oxidation state (+2, + 3, and +4), spin state, and Ni–O bond length. The dynamic redox behavior of Ni ions in different reactions has been found to be crucial for OER and other anodic oxidation processes. Therefore, designing efficient Ni-based electrocatalysts for anodic oxidation reactions requires a comprehensive understanding of their structure–activity relationships and underlying reaction mechanisms. In this perspective, the mechanistic aspects of different anodic reactions with Ni-based catalysts have been described with the help of in situ and ex situ spectroscopic, microscopic, and analytical techniques. Further, theoretical calculations have been introduced for a clearer understanding of the structure–activity relationship.

## Graphical abstract

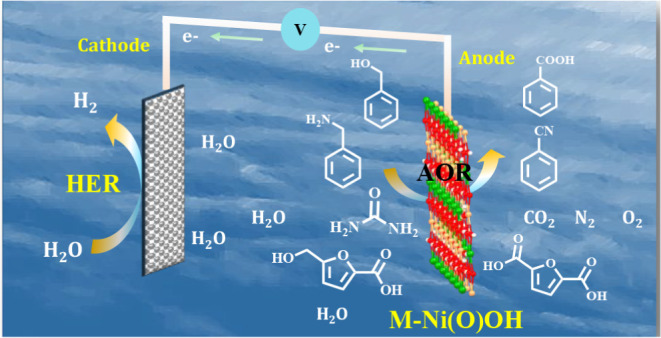

## Introduction

Among different 3d transition metal electrocatalysts, Ni-based oxides, hydroxides, perovskites, spinels, phosphides, nitrides, chalcogenides, metal–organic frameworks (MOFs), molecular complexes, coordination polymers (CPs), etc., show remarkable activity in anodic oxygen evolution reaction (OER), organic oxidation reaction (OOR), biomass valorization, plastic upcycling, and small-molecule activation processes.^1–3^ All the aforementioned catalysts undergo in situ anodic reconstruction in an alkaline medium to produce the active phase Ni­(O)­(OH).^1,4^ Depending on the nature of the precatalyst and the applied anodic potential, the reconstruction can occur either at the surface of the particle or throughout the bulk material.^5–7^ During the anodic reconstruction, the catalysts undergo a series of changes, including variations in chemical composition, crystal phase, electronic structure, electrochemical surface area, metal–oxygen bond distance, etc. (Figure 1).^8,9^ In addition, selective leaching of the cation and/or anion also produces a defect-rich structure, including cationic, anionic, and/or both vacancies.^10,11^

**Figure 1:**
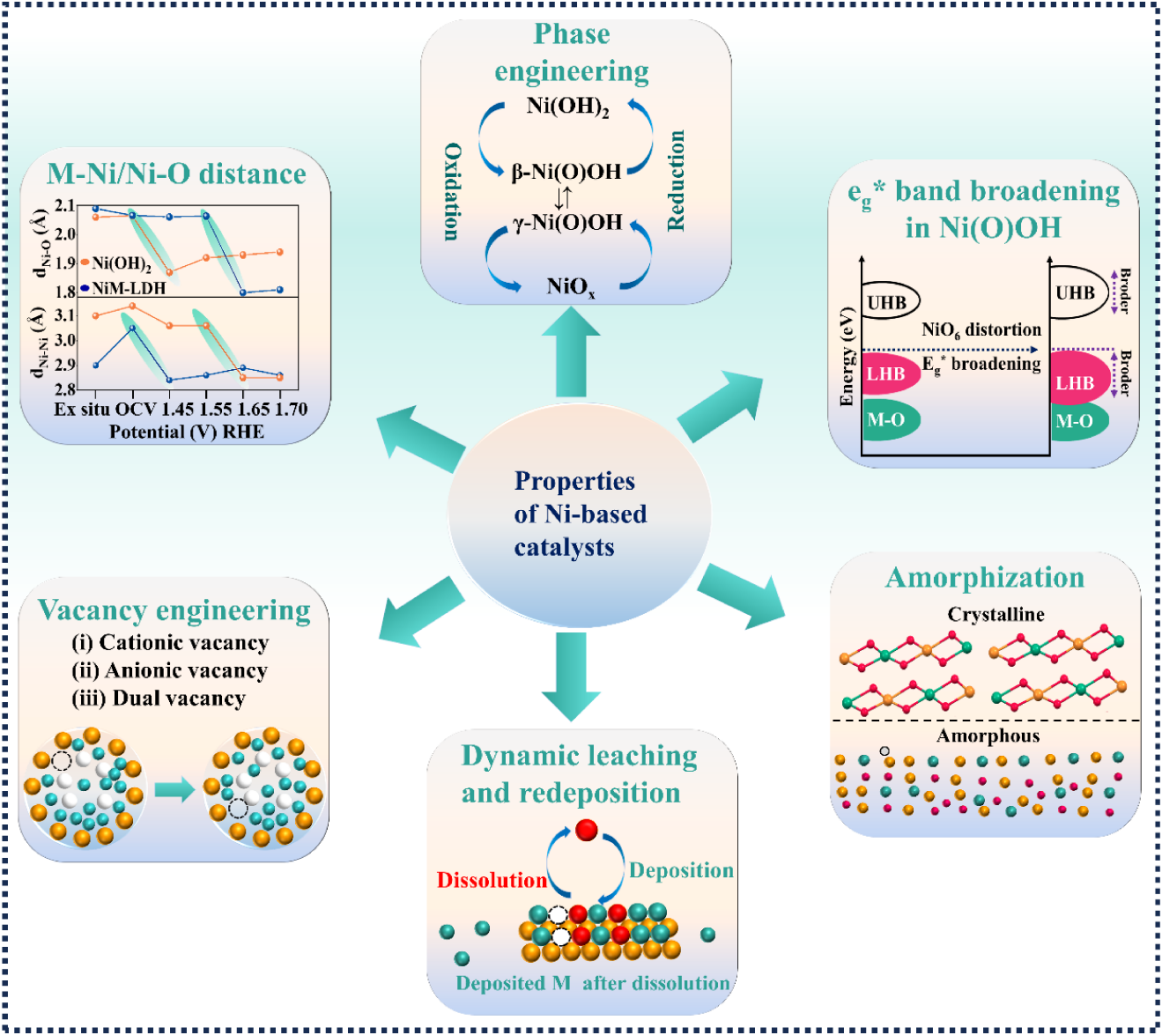
OER descriptors for the active catalyst Ni­(O)­OH formed by the anodic activation of Ni-based precatalysts.

The design of high-performance Ni-based OER catalysts relies on three key factors: (i) reconstruction into the active Ni­(O)­OH phase, (ii) accessing more active sites, and (iii) fine-tuning of the electronic structure (Figure 1).^12,13^ These targets can be achieved through doping or substitution in the catalyst structure, defect engineering, strain and phase modulation, and construction of heterostructures.^14,15^

Heteroatom doping modifies the electronic structure, generates defects, and stabilizes a higher oxidation state of Ni to boost the activity.^16–19^ To provide an in-depth understanding of heteroatom incorporation in the structure of Ni­(O)­OH, we have chosen four multivalent 3d metal ions (Mn, Fe, Co, Cu) and elucidated their influence on the electrocatalytic performance of the active catalysts.^17,18,20^ Among the different dopants, Fe is particularly effective in enhancing the OER activity of Ni­(O)­OH by facilitating charge transfer and producing new active sites. However, the presence of excess Fe in the catalyst structure can lead to the separation of the Fe­(O)­OH phase, reducing overall activity and stability. Further, Fe-rich phases suffer from higher dissolution of Fe compared to Ni or Co analogues, leading to a compromise between activity and long-term durability.^17,18,20^ Selective leaching and redeposition of Fe from Fe–Ni­(O)­(OH) can help maintain high activity through dynamic surface reconstruction.

Under anodic conditions, Ni^2+^ is oxidized to higher oxidation states like Ni^3+^ and Ni^4+^, favoring the adsorption and activation of oxygen intermediates.^21–23^ In situ X-ray absorption spectroscopy (XAS) analyses (including X-ray absorption near-edge spectroscopy (XANES) and X-ray absorption fine structure (EXAFS)) of Ni-based catalysts during OER revealed the formation of Ni­(O)­OH, having high-valent Ni^3+^/Ni^4+^ species with shorter Ni–O and Ni–Ni distances (Figure 1).^21,22^ The high positive charge density of Ni^4+^ in Ni­(O)­OH generates stronger electrostatic interactions with the intermediates (*OH, *O, and *OOH), lowering the energy barriers for the successive proton-coupled electron transfer (PCET).^24^

The density functional theory (DFT) also predicts the superior performance of Ni-based catalysts for OER over other 3d-transition metals (TM) because of a favorable interaction of the reaction intermediates with the active phase.^25,26^ After the doping of other metals, such as Co and Fe, in Ni­(O)­OH, the partial density of states (PDOS) of Ni–O 2p orbitals reaches near the Fermi level, enhancing the electronic conductivity and the adsorption of OER intermediates. For example, the substitution of Ni with Fe in Ni­(O)­OH shifts the O 2p band center closer to the Fermi level, resulting in a notably improved OER activity.^25^

Recently, NiFe-LDHs and CoFe-LDHs have been widely explored for OER. However, NiFe-LDH shows a superior performance due to stronger 3d–O 2p hybridization, shorter Fe–O bonds, stronger Fe–Ni interactions, and the redox flexibility of Ni-ion (Ni^2+^, Ni^3+^, Ni^4+^).^27–29^ In contrast, CoFe-LDH has weaker Co–Fe coupling and less efficient charge delocalization, resulting in a lower activity.

The replacement of OER with thermodynamically favorable electrochemical oxidation reactions has emerged as an energy-saving and cost-effective strategy. The unique redox behavior and the versatility of Ni-based catalysts have been explored for the electrooxidation of organic compounds such as alcohols, carbonyls, urea, biomass, and amines to value-added chemicals.^30,31^ In contrast to OER, during the electrochemical oxidation of organic molecules, the Ni^3+^ species in β-Ni­(O)­OH function as a redox mediator, providing active sites to abstract hydrogen atoms from the organic substrates without significantly involving the lattice oxygen.^30,32^ In OOR, the presence of Ni^4+^ or the involvement of lattice oxygen is not desirable, as it leads to overoxidation and the formation of undesired byproducts, reducing the faradaic efficiency (FE).^30^

Notably, the number of publications on Ni­(O)­OH for the OER and other anodic reactions has surged since 2013. In this context, this perspective will uncover the following points: (i) the electrocatalytic activity of Ni-based materials for OER, OOR, biomass oxidation, and plastic valorization; (ii) the impact of the electronic structure modulation of Ni in the active catalyst for improved activity; (iii) the major challenges associated with catalyst design; and (iv) potential applications of Ni-based materials in electrocatalytic anodic processes.

## Ni-Based Catalysts for OER

The OER proceeds through three different pathways: (i) the adsorbate evolution mechanism (AEM), (ii) the oxide path mechanism (OPM), and (iii) the lattice oxygen mechanism (LOM) (Figure 2a–c).^33,34^ The AEM proceeds through a series of concerted proton–electron transfer steps involving surface-bound intermediates, leading to O–O bond formation without inducing any lattice distortion.^35,36^ In contrast, the LOM is followed when two interconnected conditions are satisfied: (i) the active catalyst, following anodic oxidation, reaches such a state of electronic saturation that a high positive charge density is generated on the oxygen ligands; and (ii) the efficiency of the catalyst lattice framework to accommodate the oxygen vacancy formed during the dynamic structural evolution and its subsequent healing.^33,37,38^

**Figure 2:**
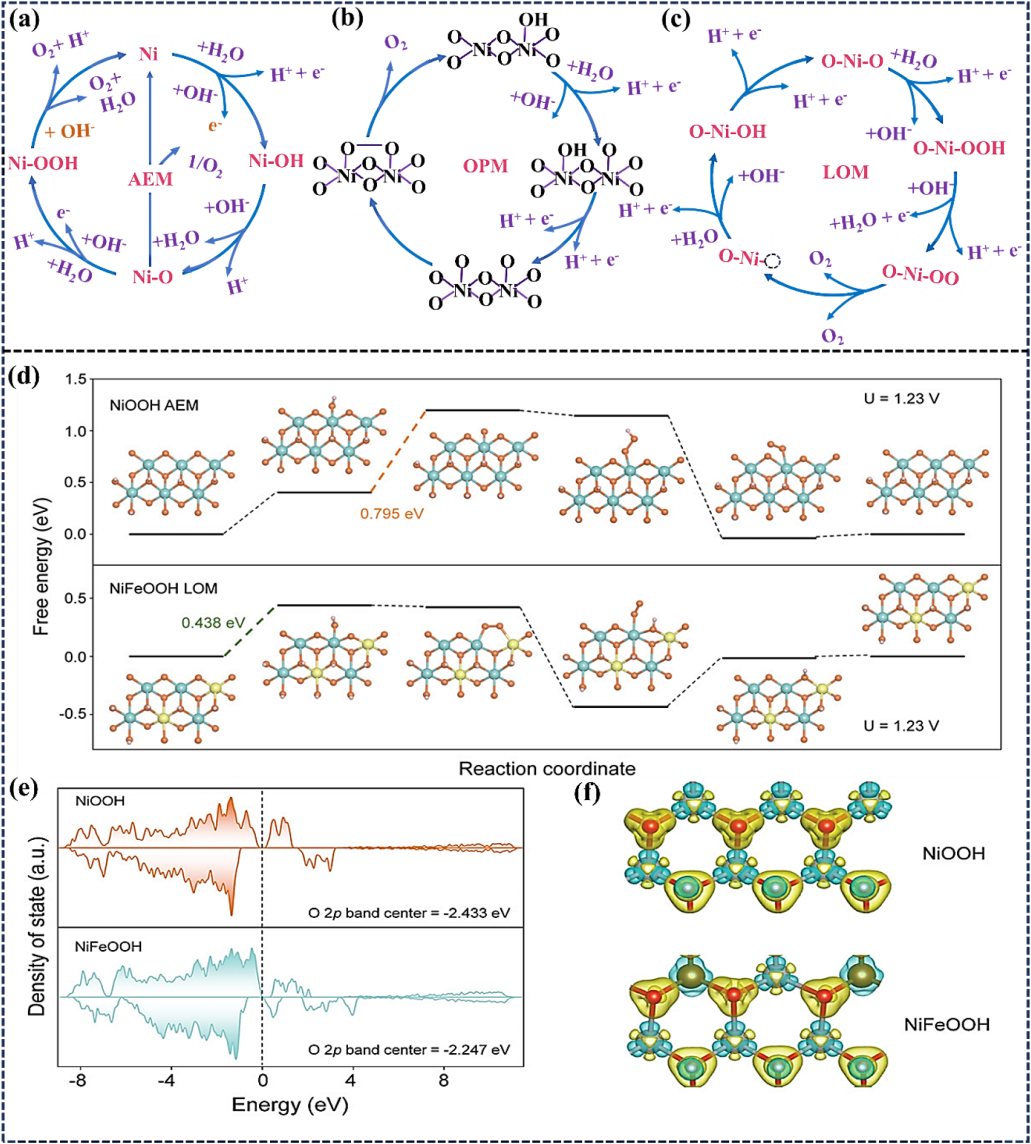
(a–c) Schematic representations of the AEM, OPM, and LOM pathways for OER. (d) Calculated OER free-energy diagrams for Ni­(O)­OH and NiFe­(O)­OH at *U* = 1.23 V for both LOM and AEM pathways. (e) PDOS and (f) charge density difference plots for Ni­(O)­OH and NiFe­(O)­OH slabs. Reproduced with permission from ref 25. Copyright 2022, Wiley.

In the OPM pathway, OER proceeds through direct O–O radical coupling without the formation of lattice defects or additional intermediates (OOH*).^39–41^ The mechanism involves only O* and OH* species as key intermediates. As a result, the intrinsic activity-stability trade-off typically observed in the AEM and LOM pathways does not exist in OPM.^39–41^

In this regard, Fe, Co, and Ni-based oxyhydroxides have been extensively studied for OER in alkaline media, specifically NiFe­(O)­OH, whose catalytic activity has surpassed that of Fe and Co-based catalysts. However, the OER activity of NiFe­(O)­OH is still severely restricted by the AEM pathway. Although the theoretical studies suggested that Fe atoms in γ-Ni­(O)­OH act as the oxygen-evolving center, assigning active centers for OPM is difficult.^39–41^

The DFT calculation suggests that the O–O coupling energy for pristine Ni­(O)­OH is very high (Δ*G* = 0.947 eV), and a significant decrease in the Δ*G* value was observed for NiFe­(O)­OH (Δ*G* = −0.017 eV), indicating that the OER follows an LOM route.^39–41^ The Fe doping in Ni­(O)­OH activates the surface oxygen atoms, enabling the reconfiguration of the reaction intermediates. Different research groups also report similar findings, demonstrating that Fe-doped Ni­(O)­OH preferentially follows LOM.

Furthermore, it was observed that the Co-doped Ni­(O)­OH showed a different OER mechanism. In this category, Patzke’s group studied the OER with Ni_3_Co_1_-CPs and proposed the OPM pathway, in which the O–O bond formation proceeds via the catalytically active Ni^4+^–O–Co^4+^ moieties.^42^

Therefore, it can be concluded that the pristine Ni­(O)­OH follows an AEM pathway of OER, while the Fe or Co doping in Ni­(O)­OH can change the mechanism to LOM or OPM.

### Monitoring Structural Evolution of Ni-Based Catalysts during OER

The electrochemical reconstruction of the Ni-catalyst in a strongly alkaline medium (pH: 13–14) has been systematically studied by CV.^14,43,44^ The potential window is divided into a low-potential region (−0.2 to 0.5 V_RHE_), where Ni­(OH)_2_ shows reversible redox behavior, and a high-potential region (0.5 to 1.6 V_RHE_), where Ni-oxo species are formed by an irreversible transition.^30^ In the high-potential region, α-Ni­(OH)_2_ is irreversibly converted into β-Ni­(OH)_2_, with the simultaneous formation of NiO_*x*_ species (0.5–1.2 V_RHE_), followed by the further transformation to γ/β-Ni­(O)­OH (>1.2 V_RHE_).^30^

Operando and in situ Raman studies detected the formation of high-valent Ni­(O)­OH species at >1.5 V_RHE_. While this nonstoichiometric Ni­(O)­OH phase governs the anodic oxidation activity, its formation mostly depends on the precursor’s structure and morphology and the process of anodic activation.^14,30^ The reconstruction of the precatalyst can be precisely tuned through heteroatom doping (e.g., Fe, Mn, Co), anion variation (e.g., O/S/Se/P), ligand engineering, and defect formation (metal and oxygen vacancies), etc.^25,26,39^ These factors collectively regulate the Ni^2+/3+/4+^ redox transitions, stabilize high-valent active species, and optimize the adsorption energies of the key intermediates. The rate and extent of reconstruction play a key role in determining the electrochemical OER activity and stability.^25,26,39^

For example, Ni_*x*_S_*y*_-based catalysts show outstanding OER activity owing to their tailored electronic structure, dynamic surface reconstruction, and fast charge-transfer kinetics. Under alkaline conditions, sulfur leaching and oxidation lead to the formation of a porous, defect-rich Ni­(O)­OH layer enriched with Ni^3+/4+^ species.^25,26,39^ The sulfur incorporation modulates the Ni d-band center, improving the adsorption energetics of *OH, *O, and *OOH intermediates. Nevertheless, over-reconstruction may damage structural stability, leading to gradual dissolution and durability loss. In contrast, phosphide precursors undergo more controlled reconstruction, forming a thin Ni­(O)­OH shell on the catalyst and preserving the electron-conductive phosphide core.^25,39^

NiSe delivers excellent OER activity due to its high metallic conductivity and dynamic surface reconstruction, forming the surface-active catalyst with Ni^2+/3+/4+^ species and a conducting selenide core. Partial Se leaching produces anionic defects and vacancies, increasing the active site exposure.^39^

During OER, NiO is transformed into Ni­(OH)_2_ and further oxidized to Ni­(O)­OH, where Ni^3+^ and transient Ni^4+^ species facilitate O–O bond formation. This redox flexibility promotes efficient electron transfer and continuous regeneration of active sites. Additionally, the layered Ni­(OH)_2_ structure enhances OH^–^ intercalation and ion transport to the catalytic surface.^39^

Ni-based LDHs undergo gradual oxidation of Ni^2+^ to Ni^3+/4+^ without severe structural collapse.^39^ Although their initial activity is less pronounced than that of sulfides or selenides, the preserved layered framework ensures a superior durability. LDHs avoid bulk degradation and achieve the best balance between activity and stability.^39^

Apart from the interlayer hydrogen bonding in Ni­(O)­OH, the diffusion of protons is additionally impeded by the Jahn–Teller (JT) distortion in Ni^3+^O_6_ octahedra (Figure 3).^39–41^ Given that the $d_{x^{2}-y^{2}}$ and d*z* ^2^ orbitals of Ni^3+^ (t_2g_^6^ e_g_^1^) are degenerate, JT distortion results in the separation of the degenerate electronic states, while Ni^2+^ (t_2g_^6^ e_g_^2^) and Ni^4+^ (t_2g_^6^e_g_^0^) do not experience this type of JT distortion.^40^

**Figure 3:**
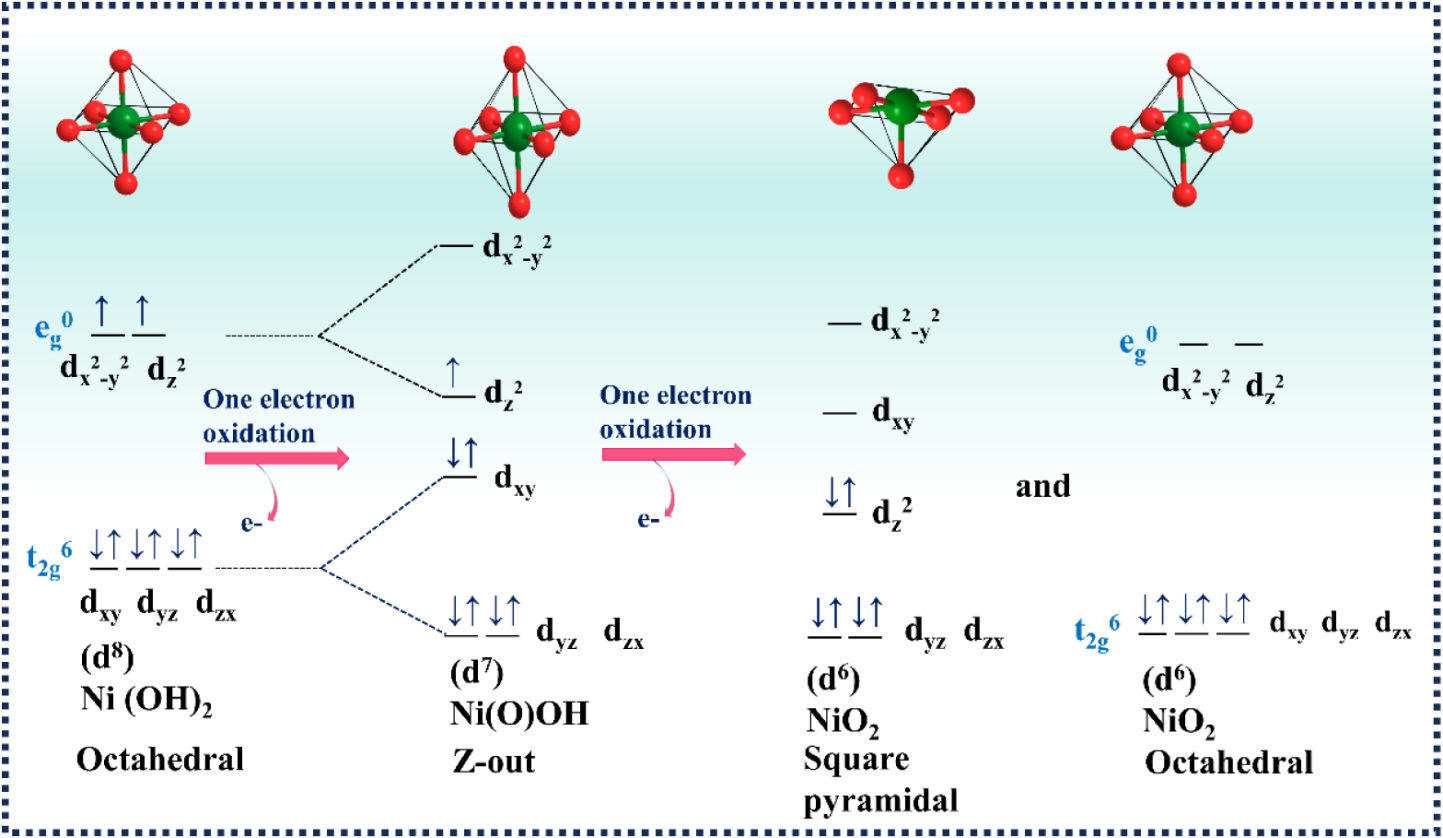
Change in the electronic configuration and geometry of Ni ions during the electrochemical transformation from Ni­(OH)_2_ to Ni­(O)­OH to NiO*_x_*.

The structural evolution of catalysts during the OER process can be monitored using a variety of in situ, operando, and ex situ techniques. After the anodic activation of the catalyst, the powder X-ray diffraction (PXRD) showed additional diffraction peaks for the Ni­(O)­OH phase.^14^ Transmission electron microscopy (TEM) can also detect the surface reconstruction during OER.^14^ The X-ray photoelectron spectroscopy (XPS) analysis of the active catalyst further confirmed the coexistence of Ni^2+^ and Ni^3+^ in it.^45^

Quasi-operando XPS study of (Ni,Mn)-(Co)_Td_(Co_2_)_Oh_O_4_ showed that an increase in the applied voltage (OCP–1.6 V_RHE_) led to a rise in the valence states of Ni and Co, as seen by a pronounced peak shift at 1.45 V_RHE_.^45^ This observation suggests that surface reconstruction occurs in the potential range of 1.40–1.45 V_RHE_. The Ni 2p XPS spectra further displayed a continuous shift toward higher binding energies, confirming the gradual increase in the Ni valence state (Figure 4a).^45^

**Figure 4:**
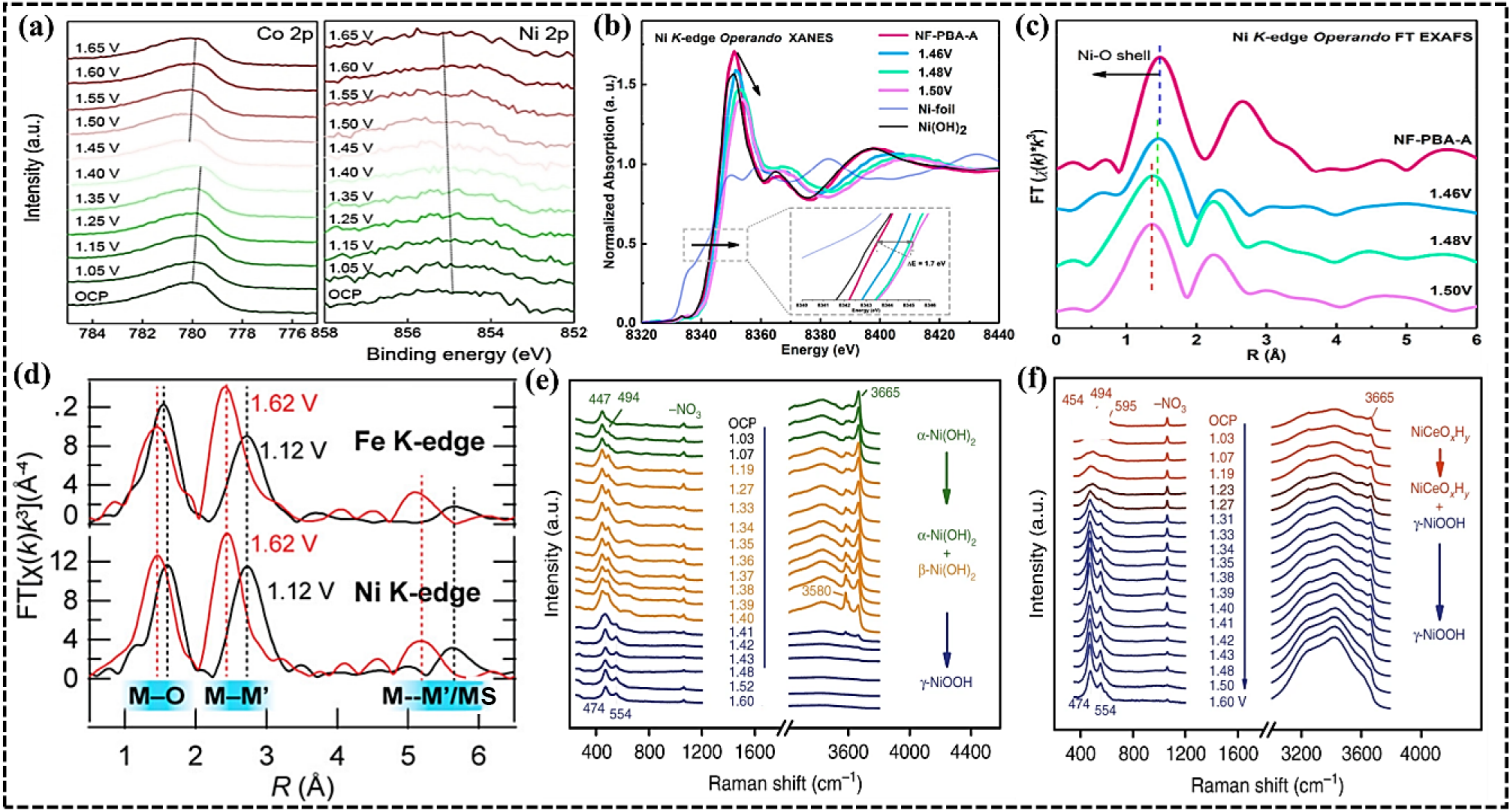
(a) Quasi-operando XPS of (Ni,Mn)-(Co)_Td_ (Co_2_)_Oh_ O_4_ showing the rise in the valence states of Ni and Co with applied potential. Reproduced with permission from ref 45. Copyright 2023, Wiley. (b) The XANES edge of NF-PBA-A at different potentials. (c) EXAFS showing a noticeable contraction in the Ni–O shell with increasing applied potential. Reproduced with permission from ref 46. Copyright 2018, American Chemical Society. (d) Operando EXAFS and Ni K-edge of NiFe­(O)­OH. Reproduced with permission from ref 21. Copyright 2015, American Chemical Society (e,f). In situ Raman spectra of (a) Ni­(OH)_2_ and NiCeO*_x_* H*_y_*. Reproduced with permission from ref 51. Copyright 2018, Nature Publishing.

The XANES and EXAFS studies of NF-PBA-A demonstrated that, after anodic activation, the electronic state of Ni in the active catalyst was more similar to that of Ni­(OH)_2_ rather than NiO_2_, suggesting that Ni does not undergo immediate oxidation upon activation (Figure 4b,c).^46^ Further operando measurements conducted at various applied potentials revealed that, below 1.5 V_RHE_, only partial dehydrogenation of Ni–OH groups occurs, resulting in the formation of mixed Ni^2+^/Ni^3+^(O)­OH. At higher potentials, successive dehydrogenation processes lead to the generation of Ni^4+^ species with NiO_2_-like characteristics, which are identified as active centers for the OER (Figure 4c).

Klaus et al. investigated the phase transition behavior of Ni­(O)­OH by controlling the Fe impurity content in the KOH solution (electrolyte).^47^ The catalyst films in purified electrolyte showed higher overpotentials, larger Tafel slopes, and poorer stability than those in KOH solution with Fe impurity. Therefore, the incorporation of Fe impurities from the KOH electrolyte is a primary factor contributing to the enhanced OER activity of Ni­(O)­OH. In situ Raman spectroscopy further revealed that films in Fe-free KOH transformed into β-Ni­(O)­OH, whereas those in Fe-containing electrolyte evolved into NiFe-LDH during aging, consistent with the observed trends in overpotentials.^47^

Although +3 is typically considered the oxidation state of Ni in Ni­(O)­OH, the formation of both Ni^3+^ and Ni^4+^ is well-known. Goldsmith et al. found that a variety of oxidation states of Ni (+2, +3, +4) and Fe (+2, +3, +4, +5) can coexist, depending on the number of hydrogen atoms in the structures.^48^

Evidence for the electrochemical stability of Fe^3+^ ions in NiFe­(O)­OH under OER conditions was provided by the Bell group.^21^ In the potential window of 1.12–1.92 V_RHE_, Fe^3+^and Ni^2+^ both oxidized to higher oxidation states (Ni^3+^, Ni^4+^ and Fe^3+^, Fe^3+δ^) as revealed by XANES (Figure 4d).^21^ Further, the work by the Strasser group with a series of Ni–Fe mixed oxides (having different Ni:Fe ratios) at a fixed potential (1.63 V_RHE_) revealed that Ni^2+^ was oxidized to Ni^3+^ and Ni^4+^, while the formal oxidation state of Fe varied between +3.1 and +3.4, as determined by the Fe–O bond lengths from EXAFS.^21^

In this category, an interesting study was reported on Ni–Bi films, where the catalytic activity increased by 3 orders of magnitude after anodic reconstruction.^49^ The XANES and EXAFS analyses of the activated film revealed a phase transition from β-Ni­(O)­OH to γ-Ni­(O)­OH in the active catalyst, accompanied by a notable increase in the population of Ni^4+^-species responsible for the enhanced OER activity. This finding challenges the previously held notion that β-Ni­(O)­OH is the superior phase for OER, suggesting instead that higher-valent Ni species in γ-Ni­(O)­OH play a more critical role in driving the reaction.^49^

Diao group compared the OER activity of β-Ni­(O)­OH, β-NiFe­(O)­OH, γ-Ni­(O)­OH, and γ-NiFe­(O)­OH.^50^ Among all the explored phases, β-NiFe­(O)­OH exhibited the highest activity with the lowest activation energy barrier in the RDS. In contrast, γ-NiFe­(O)­OH displayed a comparatively higher activation energy barrier than β-NiFe­(O)­OH.

Therefore, it can be concluded that although β-Ni­(O)­OH has a lower oxidation state than γ-Ni­(O)­OH, the optimum Ni–O bond order, higher density of hydroxyl groups, stable phase structure, and lower activation energy barrier make it a more effective OER catalyst than γ-Ni­(O)­OH.

Furthermore, Raman spectroscopy was used to identify the intermediate species, the coordination environment around the metal centers, and the active catalytic sites. For example, in Ni­(OH)_2_, the Ni–O vibrational band shifts from 447 to 463 cm^–1^ at OCP, reflecting changes in the local coordination environment.^51^ As the applied potential increases (1.41 V_RHE_), characteristic Raman bands confirm the transformation of Ni­(OH)_2_ into γ-Ni­(O)­OH. In contrast, NiCeO_*x*_H_*y*_ exhibited the formation of γ-Ni­(O)­OH phase at a much lower potential of 1.27 V_RHE_, showing that the Ce incorporation facilitated the Ni^2/3+^ transition to lower the OER overpotential.^51^ Ni, with a lower valence state in β-Ni­(O)­OH, contains a higher amount of hydroxyl groups than in γ-Ni­(O)­OH, leading to a large difference in the Raman intensity of the E_g_-band.^50^

Moreover, the quantitative correlation between the Raman characteristic changes of reaction intermediates (e.g., *OH, *O, *OO) and the dynamic evolution of active species (Ni^3+/4+^), was studied in situ for NiFeP.^38^ No significant change in the preoxidation potential was observed from open circuit voltage to 1.35 V_RHE_. As the potential increases to 1.36–1.4 V, a peak at 618 cm^–1^ appears, and its intensity first increases (1.36 V_RHE_) and then gradually weakens with increasing potential (1.41 V_RHE_).

The anodic activation of NiFeP in H_2_O and D_2_O revealed the Raman peak shift from 618 to 435 cm^–1^ due to the isotopic replacement of the *OH with *OD.^38^ The peak intensity of *E*_g_ was weaker than that of *A*_1g_ across the entire potential range, which demonstrated that the β-Ni­(O)­OH was stable under reaction conditions. Meanwhile, the Raman bands for Ni-OO species (1002 and 1034 cm^–1^), appeared in the potential range 1.41–1.5 V_RHE_, did not show any change in the peak position when H_2_O was replaced by D_2_O. In contrast, the isotopic study using H_2_^18^O showed the shifting of the peaks to 954 and 984 cm^–1^.

Furthermore, the spin state of the surface atoms plays a critical role in electrochemical reactions. According to crystal field theory, for the NiO_6_ octahedra in Ni­(O)­OH, the d orbitals of the Ni ion split into two energy levels: t_2g_ and e_g_*.^52^ The doping of Fe^3+^ (d^5^ system) in Ni­(O)­OH can make a substantial change in the local electronic structure of the catalyst, as the Fe ion can adopt a high-spin (HS) or low-spin (LS) electronic configuration depending on the crystal field splitting energy. Understanding the spin state of Fe is thus vital to unraveling its role in enhancing the OER activity of Ni­(O)­OH. Most computational studies of Fe-doped Ni­(O)­OH phases consistently report LS Ni^3+^ and HS Fe^3+^ states.^50,52^

The optimum electron occupation of the metal e_g_* orbital to attain the best OER activity was found to be 1.2.^53^ However, Ni^2+^ species with a d^8^ electronic configuration show overoccupation in the e_g_* orbitals. The presence of a second metal ion, such as Co, in Ni­(O)­OH leads to double charge exchange, where under anodic conditions, Co^2+^ is first oxidized to Co^3+^, followed by the exchange of the electron with Ni^2+^ to form Ni^3+^(t_2g_^6^e_g_^1^) which improves the OER activity.^53^

In addition, e_g_* band broadening in Ni­(O)­OH improves electrochemical OER performance because of the distortion in the NiO_6_ octahedron, leading to a downshift of the d-band and consequently improving the mobility of electrons.^54^ Ni­(O)­OH accessed through the anodic activation of NiS_2_, NiSe_2_, Ni_5_P_4_, and Ni­(OH)_2_ followed the OER trend Ni­(OH)_2_ < Ni_5_P_4_–Ni­(OH)_2_ < NiSe_2_–Ni­(OH)_2_ < NiS_2_–Ni­(OH)_2_, similar to the e_g_* band broadening (Figure 5a–e).^54^

**Figure 5:**
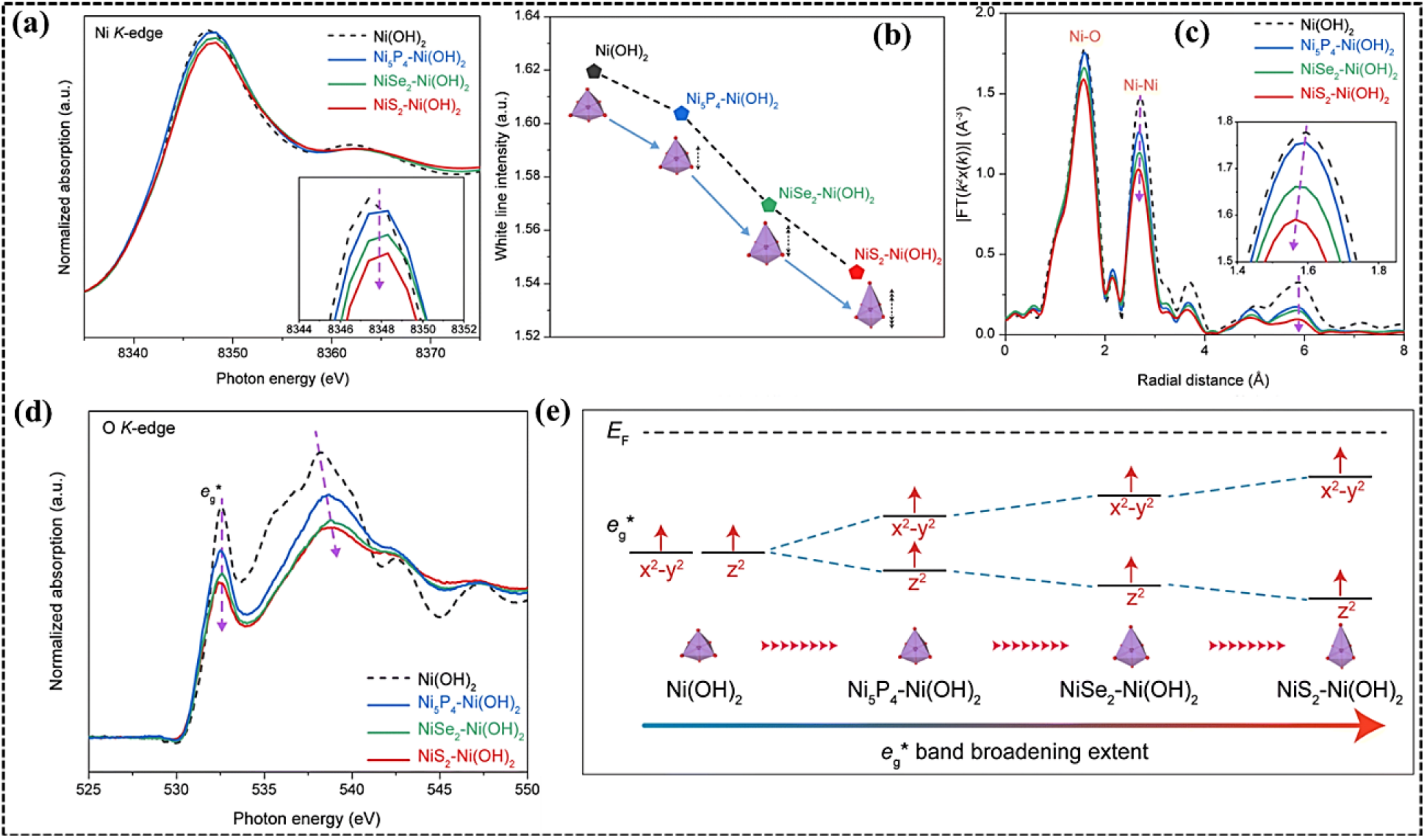
(a) Ni K-edge XANES spectra of X-Ni­(OH)_2_ (X = NiS_2_, NiSe_2_, Ni_5_P_4_). (b) The reduced white-line intensity (from figure a) showing an increased distortion in the NiO_6_ octahedron. (c) Ni K-edge FT-EXAFS of X-Ni­(OH)_2_ catalysts. (d) Normalized O K-edge XANES spectra of X-Ni­(OH)_2_. (e) Distortion in NiO_6_ octahedral by e_g_* band broadening. Reproduced with permission from ref 54. Copyright 2023, Royal Society of Chemistry.

The NiS_2_–Ni­(OH)_2_ shows the lowest white-line intensity, followed sequentially by NiSe_2_–Ni­(OH)_2_, Ni_5_P_4_–Ni­(OH)_2_, and pristine Ni­(OH)_2_ (Figure 5a).^54^ The rising absorption edge and white-line feature originate from electronic transitions from the Ni 1s to 4p orbitals. In NiO_6_ octahedral coordination, the white-line intensity is sensitive to local geometric distortion, where increased distortion typically manifests as a broader and less intense white line (Figure 5a,b). Accordingly, the degree of NiO_6_ octahedral distortion progressively increases in the order of Ni­(OH)_2_ < Ni_5_P_4_–Ni­(OH)_2_ < NiSe_2_–Ni­(OH)_2_ < NiS_2_–Ni­(OH)_2_. The increased distortion in the NiO_6_ octahedron was further examined using FT-EXAFS spectra (Figure 5c).

Among all the catalysts, NiS_2_–Ni­(OH)_2_ shows the lowest Ni–O coordination peak intensity and the shortest radial distance, following the order: NiS_2_–Ni­(OH)_2_ < NiSe_2_–Ni­(OH)_2_ < Ni_5_P_4_–Ni­(OH)_2_ < Ni­(OH)_2_.^54^ The attenuated Ni–O peak intensity further corroborates the enhanced structural distortion of the NiO_6_ octahedron. However, in the O K-edge XAS spectra, no visible peak was observed at ∼529.5 eV, indicating that the influence of oxygen vacancies on the catalytic performance can be neglected (Figure 5d). Furthermore, the absence of a characteristic band at ∼590 cm^–1^ for X–NiOOH (X = NiS_2_, NiSe_2_, Ni_5_P_5_) confirms that cation vacancies are also not present in these materials.

A similar trend was reported by Zhong et al. in the Fe-doped NiOOH.^39^ They found that Fe doping effectively modulates the NiO_6_ octahedral distortion in Ni­(O)­OH, resulting in varying degrees of e_g_* band broadening. An increased e_g_* bandwidth markedly accelerates OH deprotonation in the low-potential region, where it serves as the RDS. At higher potentials, stronger e_g_* band broadening enlarges the nonoverlapping region between the d*z* ^2^ and a_1g_* orbitals, which significantly enhances photon utilization by promoting photon-induced electron transfer from M–O bonds to unoccupied d*z* ^2^ orbitals. Consequently, under the coupled oxygen evolution mechanism, catalysts with a higher degree of e_g_* band broadening exhibit a more pronounced enhancement in OER activity.^39^

### Seawater Electrolysis

Compared to freshwater electrolysis, seawater splitting remains highly challenging due to its complex composition, especially the high concentration of Cl^–^ ions (about 0.5 M), which leads to the competing Cl_2_ evolution reaction (ClER). The evolved Cl_2_ also corrodes the electrode and the electrochemical cell.^55–57^ In addition, the abundant Ca^2+^ and Mg^2+^ ions in seawater can lead to the deposition of corresponding hydroxides on the electrode surface, poisoning the electrocatalysts and significantly reducing the catalytic activity.^55–60^

Under alkaline conditions (pH > 7.5), the potential difference between OER and ClER is 480 mV, which offers a convenient potential window to avoid Cl_2_ evolution during the electrolysis of seawater. However, even the most active Ni-based electrocatalysts cannot reach a desired high current density at an overpotential below 480 mV.^55–60^ Although Ni-based catalysts showed high OER activity in fresh water, their activity dramatically decreases in seawater.^60^

For the moment, the problem of electrocatalyst design for seawater splitting mainly lies in improving its corrosion resistance and oxygen evolution selectivity; the high concentration of chloride anions can corrode metallic catalysts and oxidize them to polluting chlorine gas or hypochlorite, resulting in a dramatic drop in electrolysis efficiency.^55–60^ Moreover, the slow intrinsic reaction rate and the diversity of interfering ions in seawater are additional design challenges.

To resolve this problem, research groups have utilized different strategies, including forming a thin protective layer to increase catalyst stability, using alloying, employing surface reconstruction engineering, and increasing conductivity.^55–60^ For example, Ma et al. demonstrated that the introduction of SO_4_^2–^ to the electrolyte led to its adsorption on the catalyst surface to repel Cl^–^ by an electrostatic repulsive force to improve the stability of Ni­(O)­OH in alkaline seawater electrolysis.^61^ Kuang et al. reported that polyatomic sulfate generated on the surface of NiS_*x*_ can also effectively repel Cl^–^ during seawater oxidation.^62^ In this category, the in situ formation of a PO_4_^3–^ oxyanion layer has been reported as an effective strategy, acting as a Cl^–^ repellent, thereby enhancing the robustness and stability of NiFe­(O)­OH during seawater oxidation.^58,60^

### Anodic Oxidation Reactions beyond Oxygen Evolution

Anodic oxidation of a wide range of small molecules, including alcohols, aldehydes, amines, urea, and various biomass-derived organic compounds, has been reported for hybrid water electrolysis.^1,63–65^ In this context, Ni-based catalysts have shown exceptionally high activity and product selectivity.

Electrochemical organic oxidation with Ni-based catalysts follows two main pathways. The classical Fleischmann mechanism involves Ni­(OH)_2_/Ni­(O)­OH redox couple, where Ni­(O)­OH oxidizes the organic substrate and is reduced back to Ni­(OH)_2_.^66^ For alcohols, the rate-determining step is the hydrogen atom transfer (HAT)-driven formation of a carbon-centered radical.^66^

In contrast, the hydride-transfer mechanism proposed by Choi and Hammes–Schiffer differentiates the subtle potential difference between the Ni redox peak and the alcohol oxidation peak.^64,66^ Linear sweep voltammetry during OOR reveals that the balance between “indirect” and potential-dependent oxidation strongly depends on the substrate structure. Without organics, a peak at ∼1.37 V_RHE_ corresponding to Ni­(OH)_2_ → Ni­(O)­OH transformation originates, followed by OER.^64^ In OOR, the Ni^3+^ to Ni^2+^ reduction via PCET is generally the RDS.^64^ In contrast, ethanol and benzyl alcohol oxidation show a minute change in this redox peak but display a new anodic wave at higher potential, indicating a different potential-dependent oxidation pathway.

Although Ni­(O)­OH is found to be the active catalyst for the anodic oxidation reactions, recent works have established that the mechanisms of OER and other OORs are completely different.^63–65^ In the case of OER, the facile formation of Ni^IV^=O and subsequent nucleophilic attack of OH^–^ are the crucial steps, while the organic oxidation reactions proceed through a chemical reaction instead of a direct electrochemical reaction.^66,67^ The Ni^3+^ species in Ni­(O)­OH oxidizes the organic molecule by the dehydrogenation method and produces Ni­(OH)_2_, which again gets oxidized to Ni­(O)­OH by the electrochemical method. The formation of Ni^IV^-species often reduces the selectivity of product formation in OOR by overoxidation, and a strong interference from OER has also been observed to reduce the FE of the desired product.

### Mechanism of Electrooxidation of Organics

Typically, after the application of an anodic potential, an electron is abstracted from the highest occupied molecular orbital (HOMO) of the substrate, leading to the formation of a radical cation.^30,68^ This highly reactive intermediate is prone to various follow-up transformations, such as dimerization, nucleophilic substitution, and the elimination of functional groups or atoms. When two different compounds possess closely aligned HOMO energy levels, the probability of electron abstraction from any of the species becomes similar.^30^ This often leads to incomplete conversion or the formation of nonselective products. To address this, the HOMO of the substrate can be modulated through appropriate functionalization, enabling oxidation at lower potentials with enhanced product selectivity.

### Alcohol Oxidation

The recent studies have extensively explored the intricacies of methanol oxidation (MO), ethanol oxidation (EO), propanol oxidation (PO), ethylene glycol oxidation (EGO), glycerol oxidation (GyO), and PhCH_2_OH oxidation, employing Ni-based catalysts.^30,69,70^ Similarly, amine oxidation includes different benzyl and aliphatic amines to form the cyano compounds.^71,72^ During alcohol and amine oxidation, Ni­(O)­OH reversibly forms Ni­(OH)_2_ species and simultaneously oxidizes the organic molecules. This mechanism can proceed via the HAT or hydride (H^–^) transfer pathway (Figure 6a,b).^69–72^

**Figure 6:**
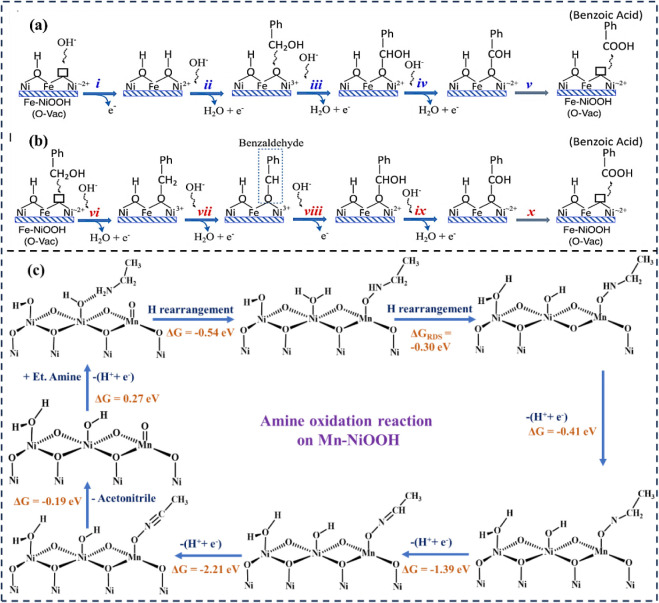
(a,b) Schematic illustration of the PhCH_2_OH oxidation mechanism on NiFe­(O)­OH, highlighting (a) redox-mediated and (b) oxygen-vacancy-driven pathways. Reproduced with permission from ref 70. Copyright 2023, American Chemical Society. (c) Proposed amine oxidation mechanism on MnNi­(O)­OH. Adapted from ref 72.

Typically, HAT is a chemical process rather than an electrochemical process, which is commonly referred to as the “indirect oxidation” (ID). Alternatively, the reaction mechanism can also proceed via potential-dependent pathways through the hydride transfer route.^69,70^ According to the Hahn group, the oxidation of alcohols proceeds mainly via two pathways: redox (indirect) and vacancy-driven mechanisms.^71^ The redox mechanism is preferred due to the favorable adsorption energy. In the redox mechanism, alcohol adsorbs on the catalyst surface, then the α-C–OH bond is deprotonated to form an intermediate species. Finally, deprotonation at the α-C position forms the carboxylic acid, with the regeneration of Ni­(OH)_2_ (Figure 6a,b).^70^

Different research groups explored a series of Ni-based catalysts for PhCH_2_OH oxidation, showing the effect of structural diversity and electronic structure modulation on the catalytic activity.^1–3,69^ The heteroatom-doped Ni­(O)­OH showed a better catalytic activity than pure Ni­(O)­OH.^69^ Further, the Co–Ni­(O)­OH combination was found to produce better benzyl alcohol oxidation activity compared to Fe–Ni­(O)­OH.^69,70^ Recently, we showed that CoNi­(O)­OH, formed by the anodic activation of Prussian blue analogue (PBA), produced far better electrochemical OER activity compared to Co­(O)­OH and Ni­(O)­OH, prepared by a similar method (Figure 7a,b).^69^ A similar trend was also observed in PhCH_2_OH oxidation (Figure 7c). CoNi­(O)­OH reached a higher current density (industrial current density at 1.38 V_RHE_), significantly higher than those of Ni­(O)­OH and Co­(O)­OH and comparable to those reported for CoNi-based catalysts (Figure 7d,e).^69^

**Figure 7:**
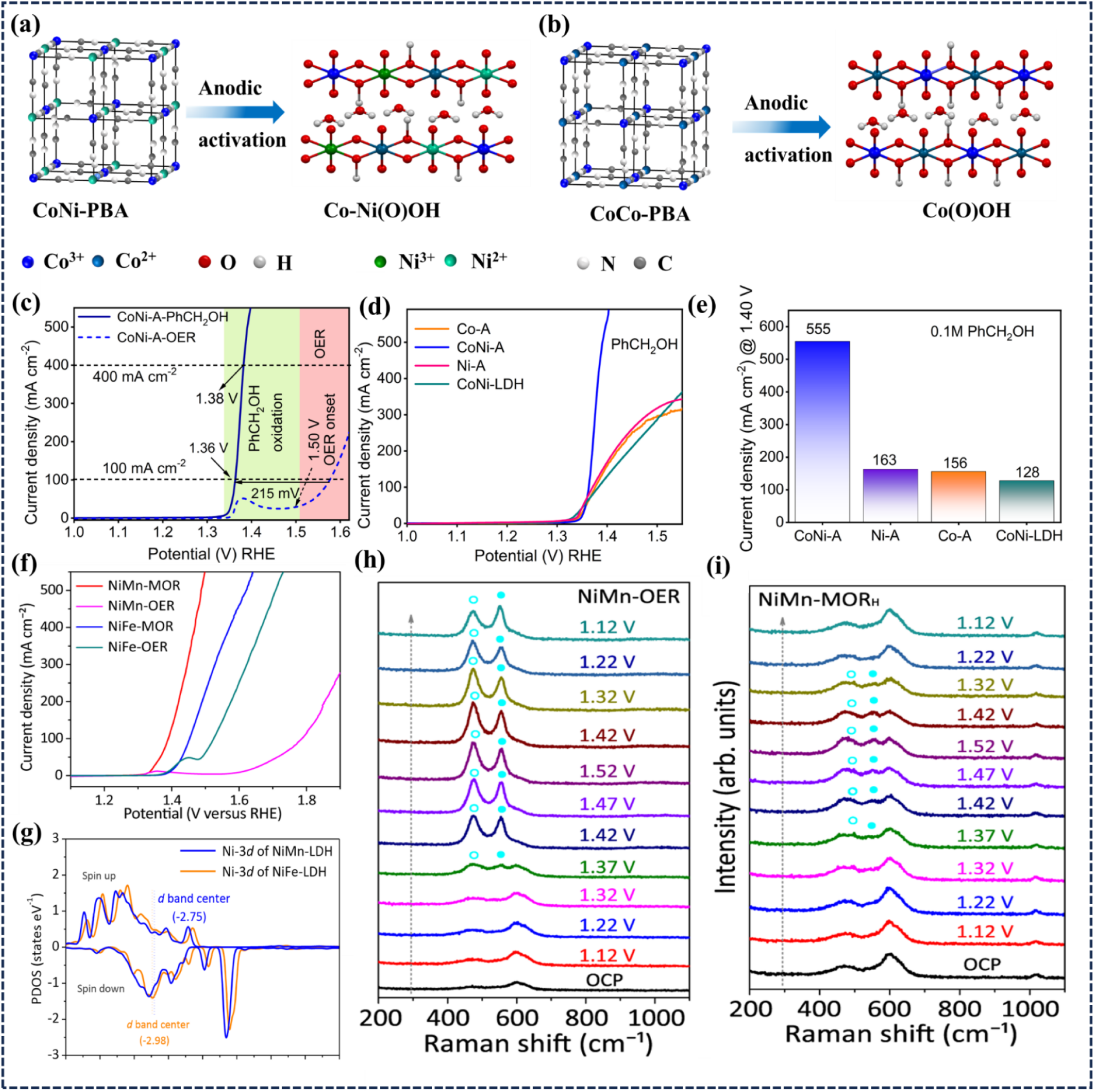
(a,b) Anodic activation of the PBA to the corresponding metal (oxy)­hydroxide, (c) LSV profiles showing the potential gain when OER is replaced by benzyl alcohol oxidation with catalyst CoNi-A, (d) LSV profiles showing the superior benzyl alcohol oxidation activity of CoNi-A compared to other catalysts, (e) Comparison of the current densities attained for benzyl alcohol oxidation with different catalysts. (a–e) Reproduced with permission from ref 69. Copyright 2025, Wiley. (f) LSV profiles for CH_3_OH oxidation with NiMn-LDH and NiFe-LDH, (g) PDOS of Ni 3d orbitals calculated for NiMn-LDH and NiFe-LDH. (h–i) Operando Raman spectra of NiMn-LDHs during OER and MOR. Reproduced with ref 82. Copyright 2024, Nature Publishing.

The transformation of PhCH_2_OH to PhCOOH is a 4e^–^ oxidation process, while PhCHO is formed as an intermediate by 2e^–^ oxidation.^73–75^ The selectivity from 2e^_–_^ to 4e^_–_^ is primarily influenced by the reaction medium, the pH of the electrolyte solution, the applied potential, the reaction time, the passed charge, and the temperature.^73–75^ Higher potential, temperature, longer time, and higher charge passing favor 4e^–^ oxidation to produce PhCOOH, while 2e^–^ oxidation products form at lower potential (1.28 V_RHE_ to 1.35 V_RHE_) and at lower temperature (20 to 30 °C).^73–75^

So far, most of the reported Ni-based catalysts offer PhCH_2_OH oxidation at higher alkaline pH (13–14), and they exhibit PhCOOH selectivity ≥90%. However, few catalysts tested in lower alkaline media have yielded the 2e^–^ oxidation product. For example, a detailed study by Wang and coworkers using NiO showed that reducing the KOH concentration from 1.0 to 0.1 M increased the PhCHO yield from 13% to 26%.^76^ This clearly indicates that higher OH^–^ concentration favors the 4e^–^ oxidation products. The Zhao group found that on Co_0.83_Ni_0.17_/A, the highest yield of PhCHO was observed after the 16 C charge, but after the 40 C charge, the amount of detected PhCHO decreased.^77^ This indicates that a higher charge passed favors the 4e^–^ oxidation products.

Furthermore, among aliphatic alcohols, MeOH oxidation has been broadly explored by using Ni-based catalysts.^78–81^ The reaction mechanism of MeOH oxidation is similar to that of PhCH_2_OH oxidation. Various catalysts, including Ni­(OH)_2_, ultrathin MnNiLDH, oxygen-vacancy-rich MnNi-MOF, MnNiS_2_, etc., have been reported for MOR.^78–81^

NiMn-LDH efficiently catalyzes methanol to formate with a low cell potential of 1.33 V_RHE_ to attain a current density of 10 mA cm^–2^ (Figure 7f). It delivered nearly 100% FE and excellent durability in alkaline media, outperforming NiFe-LDH.^82^ The higher activity of NiMn-LDH arises from its lower PDOS of Ni-3d electrons compared to NiFe-LDH (Figure 7g).

Operando Raman study with NiMn-LDH revealed the formation of Ni^3+^–O (551 cm^–1^) species under OER conditions, and the addition of MeOH led to a decrease in the intensity of the Raman band, suggesting differences in intermediate stability and surface coverage during the organic oxidation process (Figure 7h–i).^82^ These findings imply that Ni ions in NiMn-LDH experience a reversible redox shift from Ni^3+^ to Ni^2+^ during MOR. On the other hand, the Mn^3+^–O band (600 cm^–1^) does not change during the course of the potential sweep, suggesting that Mn ions are not involved in the redox process during MOR.

### Amine Oxidation

Similar to alcohol, the oxidation of benzylamine proceeds via Ni^3+^ species through a hydrogen rearrangement and deprotonation-oxidation pathway.^72^ In the case of Mn–Ni­(O)­OH, the amine binds to the Mn sites, and the surface hydroxyl groups facilitate a stepwise deprotonation-oxidation process, leading to the formation of the amine-to-nitrile product along with the regeneration of Mn–Ni­(OH)_2_ (Figure 6c).^72^ Interestingly, for benzylamine, mostly the four-electron oxidation product formation was observed. This is likely because the two-electron oxidation intermediate (imine) is highly unstable under the reaction conditions and, therefore, difficult to isolate from the reaction medium.^83–85^

Ding’s group explored a range of transition-metal (Mn, Fe, Co, and Cu)-doped α-Ni­(OH)_2_ and applied them for the electrooxidation of benzylamine (BA) to benzonitrile.^72^ The Mn doping in α-Ni­(OH)_2_ was found to produce the best results, achieving >99% conversion and over 96% FE. Potential-dependent (0.9 to 1.5 V_RHE_, in 0.05 V intervals) in situ Raman spectroscopy revealed the transformation of α-Mn-Ni­(OH)_2_ to Ni­(O)­OH above 1.15 V_RHE_ and the involvement of the Ni^3+/2+^ redox centers in the amine oxidation process. Interestingly, the structural involvement of Mn^3+^ is evident but occurs without any redox activity. Notably, the ν­(O–O) peak appeared after 1.45 V_RHE_, which suggests a higher barrier for Ni-OO* formation after Mn doping in α-Ni­(OH)_2_, offering a broader potential window for the surface oxygenated active species (prior to Ni–OO* and consequent OER) to participate in PhCH_2_NH_2_ oxidation. The Mn doping in α-Ni­(OH)_2_ significantly modified the amine adsorption sites and reduced the Δ*G* for deprotonation, resulting in a superior performance with >99% conversion and over 96% FE.

In situ time-resolved Raman studies during OER and benzylamine oxidation with NiO/NC (NC=N-doped carbon) revealed the rapid formation of γ-Ni­(O)­OH by anodic activation and its consumption in the presence of amine, confirming that amine oxidation proceeds through an indirect pathway mediated by γ-Ni­(O)­OH.^86^

Interestingly, in the case of amine oxidation reactions, the formation of Ni–N bond is quite important; it significantly altered the electronic properties of the nickel active sites. Both experimental data and the DFT calculations showed that the formation of these Ni–N bonds caused an upward shift of the Ni d-band center. This shift enhanced the electrophilicity of the Ni sites, making them more effective at attracting electron-rich molecules. As a result, BA molecules were adsorbed strongly on the catalyst surface to facilitate the subsequent dehydrogenation steps in BA oxidation. This electronic tuning lowered the activation energy for breaking C–H and N–H bonds, thus accelerating the reaction.

### HMF Oxidation

5-Hydroxymethylfurfural (HMF) is a versatile biomass-derived chemical with significant potential for the synthesis of a broad range of high-value products (Figure 8).^30,87–89^ In HMF oxidation, electrolyte pH plays a decisive role in determining the product selectivity. Under weakly alkaline conditions (pH < 13), oxidation initiates at the alcohol group of HMF, forming 2,5-diformylfuran (DFF), and subsequent oxidation steps yield 5-formyl-2-furancarboxylic acid (FFCA).^30,87–89^ In contrast, under strongly alkaline conditions (pH > 13), HMF is converted into 5-hydroxymethyl-2-furancarboxylic acid (HMFCA) and is further oxidized to 2,5-furandicarboxylic acid (FDCA). These observations highlight the critical influence of pH on the HMFOR pathway, and a pH value of around 8 is often recommended by most studies to achieve controlled and selective oxidation (Figure 8).^30,87–89^

**Figure 8:**
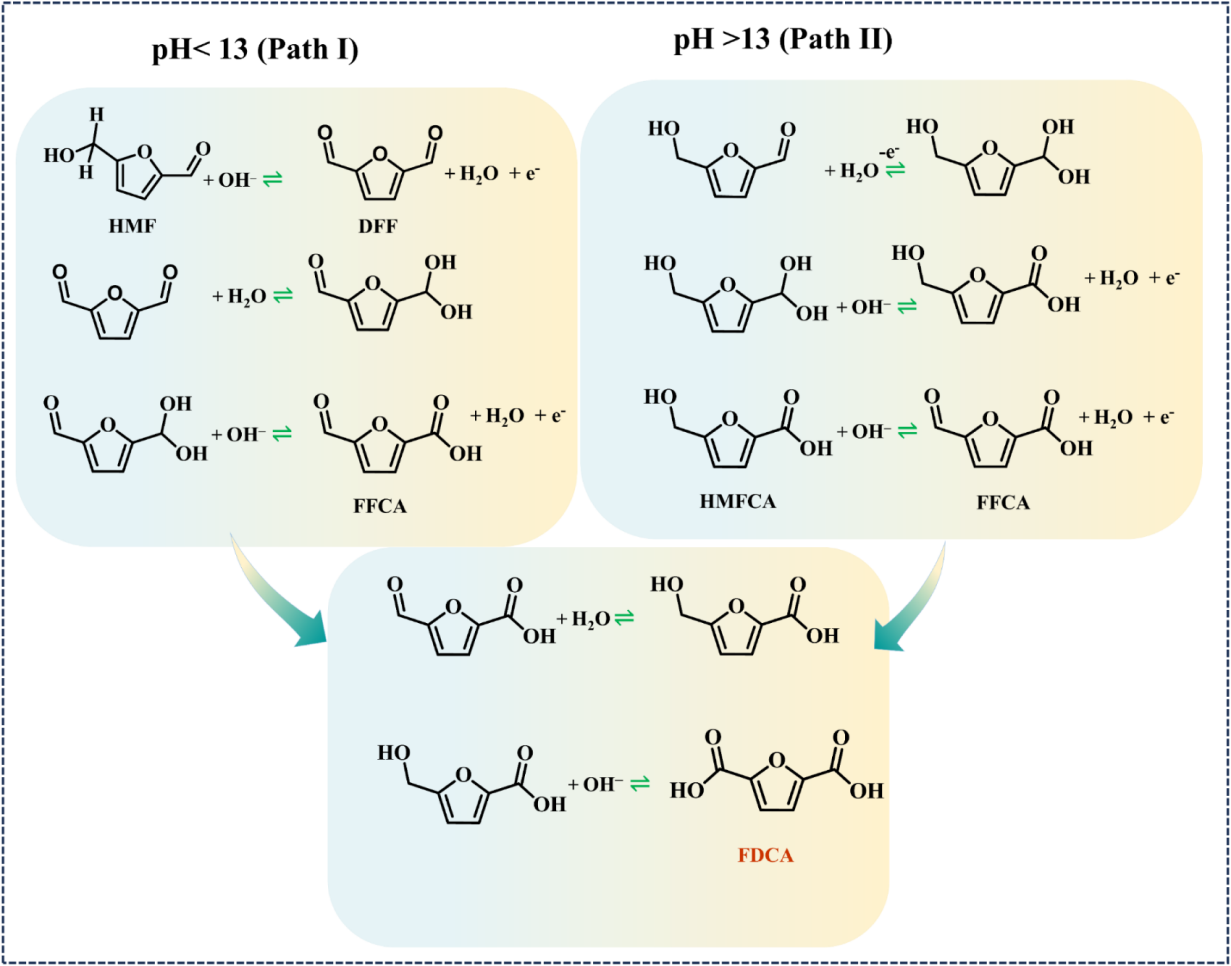
PH-dependent routes of electrocatalytic HMF oxidation to form FDCA. Adapted from ref 30.

Under strongly alkaline conditions, the HMF oxidation on Ni-based electrocatalysts is most commonly described by the indirect oxidation mechanism (IOM).^87^ In this mechanism, the applied potential drives the oxidation of Ni^2+^(OH)_2_ to the catalytically active Ni^3+^OOH species. The high-valence active sites are typically produced at relatively high potentials (1.35–1.55 V_RHE_) at high pH. During HMFOR, HMF is oxidized by the Ni^3+^ species, which are concomitantly reduced back to Ni^2+^. Notably, this oxidation pathway requires a prior activation step involving the hydration of HMF functional groups, facilitated by OH^–^ ions.

The C–H and O–H bonds associated with each functional group are subsequently deprotonated at the electrode’s active sites. The Ni­(O)­OH species function as the oxidizing agents and facilitate alcohol oxidation through a rate-limiting hydrogen atom transfer from the α-hydrogen of the alcohol group to the Ni^3+^ centers of Ni­(O)­OH, following the classical mechanism proposed by Fleischmann et al.^64,75^

The IOM is equally applicable to aldehyde oxidation, as corroborated by multiple reports.^87,89^ Once the applied potential is sufficiently high to induce rapid electrooxidation of Ni­(OH)_2_ to Ni­(O)­OH, the HMF oxidation rate becomes largely independent of potential and proceeds via a Langmuir–Hinshelwood mechanism, arising from the competitive adsorption of HMF and OH^–^ ions on the Ni­(O)­OH active sites.^64,87^

The current research to develop Ni-based catalysts for the electrochemical oxidation of HMF focuses on enhancing the selectivity of FDCA formation.^87–89^ Luo et al. studied the surface reconstruction behavior of NiFe-P under both HMF oxidation and OER conditions.^87^ The Ni­(OH)_2_–NiOOH/NiFeP heterojunction showed exceptional HMFOR activity, achieving >99% FDCA yield and >94% FE. The Yan group reported ultrathin trimetallic NiCoFe-LDH nanosheets, demonstrating superior activity in OER and HMF electrooxidation due to their reduced thickness compared to NiFe- and NiCo-LDHs.^87^ Further, Poerwoprajitno et al. improved HMF oxidation activity by using elongated branched Ni nanoparticles, with longer branches (155 ± 22 nm) showing higher specific activity.^90^ Lu et al. proposed a cobalt-doping strategy to boost the catalytic activity of β-Ni­(OH)_2_ for HMF oxidation.^91^ Raman studies demonstrated that a moderate level of cobalt doping effectively tuned the electronic structure of Ni ions, promoting the formation of catalytically active Ni^3+^–O species. Li and coworkers reported amorphous Fe/Ni­(O)­OH–SO_*x*_ from Ni_3_S_2_ (Fe/NiOOH–Ni_3_S_2_) via an iron-solution impregnation, followed by the anodic reconstruction process.^92^ The presence of SO_*x*_ species adsorbed on the surface of amorphous nickel oxyhydroxide generates unsaturated active sites, thereby promoting HMF adsorption and facilitating the oxidation reaction.

### Urea Oxidation

Among various anodic reactions, the urea oxidation reaction (UOR; thermodynamic cell potential of 0.37 V) provides a more energetically favorable route for electron supply to the coupled cathodic HER than the OER (1.23 V).^93–98^ Recent studies have clearly demonstrated that Ni plays a pivotal role in UOR catalysis.^93–98^ Ni-based catalysts exhibit high activity because the Ni^3+^ species facilitate the facile formation of a bridged coordination between the nitrogen atoms of urea and the metal sites of the catalyst. This bridge enhances the adsorption of urea on the catalyst surface and promotes its oxidation. Moreover, the redox potential of the Ni^2+^/Ni^3+^ couple in alkaline media is nearly identical to the urea electrolysis potential, which further favors efficient UOR kinetics and leads to enhanced catalytic performance.^93–98^

The six-electron UOR typically proceeds via two pathways: LOM and AEM.^93–98^ In general, the LOM pathway, involving the lattice oxygen from Ni­(O)­OH, proceeds faster than AEM. Upon activation, the catalyst surface interacts with its lattice oxygen, enabling reactions with intermediates that produce oxygen vacancies during UOR. These vacancies are subsequently refilled by OH^–^ from the electrolyte, completing the UOR cycle. However, urea oxidation may also generate byproducts such as NO_3_^–^ and NO_2_^–^, arising from preferential C–N bond cleavage.^99,100^

The coexistence of direct and indirect pathways in the UOR has been consistently supported by the mechanistic studies of various Ni-based catalysts. For example, Ni_2_P exhibited faster UOR kinetics than Ni­(OH)_2_ because of a dominant contribution from the direct UOR mechanism.^98^ This inference was drawn from a detailed analysis of the Ni^3/2+^ reduction peak in the cyclic voltammetry, which suggested a more facile catalytic pathway for Ni_2_P.^98^

Mostly, the Mn ion does not directly participate in the UOR process with the Mn-doped Ni catalyst. However, when Mn is doped with a more redox-active (redox potential range = 1.3 to 1.4 V_RHE_) metal ion, it modulates the electronic structure, tunes the occupancy of the e_g_* orbitals and d-bands, and lowers the energy barrier for the key intermediates, thereby enhancing the overall electrochemical activity.^90^ For example, Mn-doped Ni_2_P demonstrates significantly higher UOR activity compared with pristine Ni_2_P, outperforming most Ni-based UOR catalysts.^102^

Furthermore, the introduction of Mn produces a defect-rich structure that exposes abundant active sites and promotes the formation of high-valent Ni species.^100^ Mn not only facilitates the oxidation of Ni but also stabilizes the in situ-generated Ni­(O)­OH phase. As a result, the optimized f-NiMn_0.22_-LDH shows outstanding UOR activity.^100^ The Zhu group explored Ni-MnO_2_ for UOR and explained that Mn primarily modulates the electronic structure.^97^

The highly conductive Co species have also been explored for UOR.^101,102^ The uniqueness of Co introduction in Ni-based catalysts lies in its facile oxidation from Co^2+^ to Co^3+^, which subsequently facilitates the UOR process.^101^ This behavior has been demonstrated by our group and others.^102^

For example, CoNi-CP-derived CoNi­(O)­OH exhibited superior UOR activity compared to Ni­(O)­OH. This improvement arises from the facile oxidation of Ni^2+^ to Ni^3+^, induced by the rapidly formed Co^3+^ species.^101^ The Pandikumar group also reached a similar conclusion for NiCo-LDH and NiMn-LDH systems, where NiCo-LDH showed higher UOR activity than NiMn-LDH due to the facile oxidation of Ni^2+^ to Ni^3+^ in the presence of Co^3+^.^102^

### Plastic Reforming

Plastics, composed of a wide range of polymeric materials, are extensively employed in both industrial and domestic applications.^103–105^ Commonly used plastics include polyethylene (PE), polyvinyl chloride (PVC), polystyrene (PS), polyethylene terephthalate (PET), and polylactic acid (PLA).^103–105^

With the annual growth in PET production and consumption, improper disposal poses significant environmental challenges.^103–105^ Consequently, there is an urgent need for efficient and sustainable upcycling strategies for PET waste. Structurally, PET contains ester linkages that can be readily hydrolyzed under strongly acidic or basic conditions.^103^ Accordingly, numerous studies have focused on the alkaline hydrolysis of PET to produce TPA and EG, followed by the conversion of these hydrolyzates into value-added chemicals.^103–105^ The oxidation of ethylene glycol into C_2_ products is similar to the oxidation of alcohols, which we have discussed previously, while the breaking of the C–C bond to form the C_1_ products follows complex mechanisms.

Duan and the group demonstrate an electrocatalytic upcycling of PET into the commodity chemicals potassium diformate (KDF) and purified terephthalic acid (TA), coupled with H_2_ production, using a bifunctional CoNi_0.25_P electrocatalyst in a KOH electrolyte.^103^ PET was first digested under alkaline conditions to yield its monomers, including TA and EG. The EG was then selectively (>90%) converted via C–C bond cleavage to formate over the anodic CoNi_0.25_P catalyst.^103^ Furthermore, PET-derived oxidation was also evaluated using CoNi_0.25_-LDH as the catalyst. However, the activity for upcycling PET into KDF and purified TA was significantly lower than that achieved with CoNi_0.25_P.

NiFe-LDH/NF displays high activity for alkaline OER, consistent with the literature, whereas it is inferior to CoNi_0.25_P/NF for breaking the C–C bond in EG to formate.^103^ This result suggests that the active catalyst for OER may not be efficient for oxidative C–C cleavage in EG. In addition, the lower activity of CoNi_0.25_(OH)_2_/NF-derived oxy­(hydroxide) and oxide compared with its phosphide (CoNi_0.25_P/NF) indicates the important role of phosphorization in improving the activity and selectivity for cutting C–C bonds in EG to formate. The controlled experiments revealed that EG was first oxidized to glycolic aldehyde, followed by a rapid oxidative C–C cleavage to formate. Meanwhile, a fraction of glycolic aldehyde was oxidized to glycolic acid, which also underwent slow C–C cleavage to formate, producing a 90.2% total yield of formate.

In addition to real plastic, the coupling of PET-derived EGOR with HER is also explored.^94^ In this context, anodic oxidation reactions that couple the electro-reforming of waste PET-derived EG into valuable C_2_ products are explored.^104–106^ For example, Mn-doped Ni­(OH)_2_ was used to explore the intrinsic electronic effects of Mn, leading to EGOR selectivity.^104–106^ Combined experimental analyses and DFT calculations demonstrated that Mn incorporation broadens the e_g_* band, strengthening the adsorption of glyoxal intermediates. This increased interaction suppresses glycolate formation while facilitating C–C bond cleavage. As a result, Mn doping significantly increases formate selectivity, highlighting the critical role of electronic band structure modulation in directing reaction pathways. The catalysts like Pd–Ni­(OH)_2_, Pt/γ-NiOOH/NF, Pt_1_–Ni­(OH)_2_ also showed high EGOR activity.^107–109^

In situ FTIR spectroscopy was employed to identify the key intermediates involved in the C_2_ pathway during EG oxidation.^106^ The peaks at 1393 and 1580 cm^–1^, observed at a potential of 0.55 V_RHE_, correspond to the symmetric and asymmetric stretching vibrations of the COO^–^ group in GA.^106^ As the potential increased, these characteristic peaks intensified, indicating the formation of GA during the oxidation of EG. The emergence of an intense peak at 1232 cm^–1^, attributed to the C–O stretching mode, further confirmed GA formation (Figure 9).

**Figure 9:**
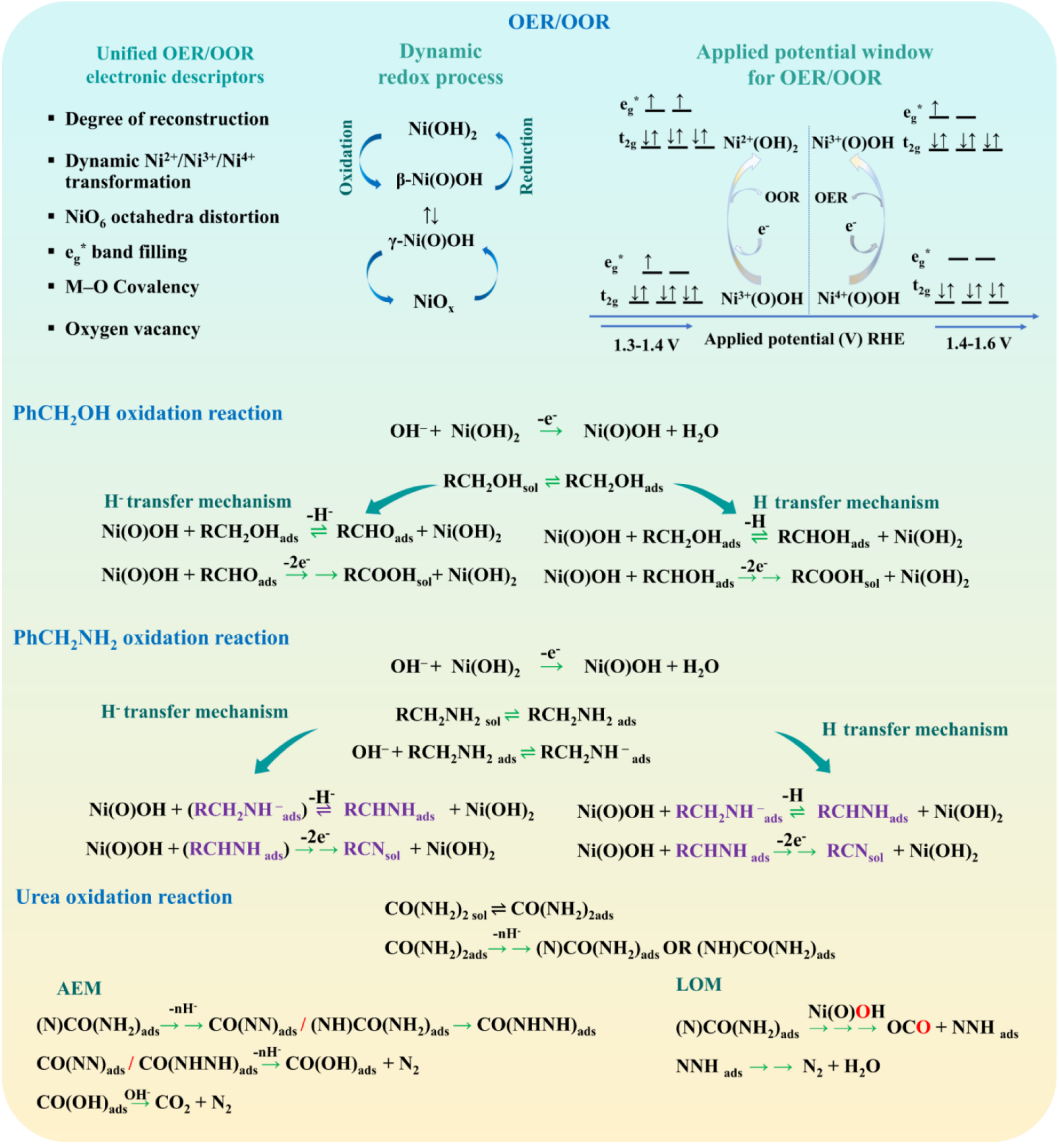
Schematic representation summarizing the key electronic descriptors leading to OER/OOR activity, associated redox dynamics, and the operative potential windows for both reactions. The illustration further outlines the mechanistic pathways and corresponding intermediates involved in the oxidation of alcohols, amines, and urea. In general, alcohol and amine oxidation proceed via either a hydride-transfer or hydrogen-atom-transfer mechanism. In contrast, the UOR predominantly follows the AEM or the LOM.

A weak peak at 1720 cm^–1^ indicated the formation of aldehyde intermediate glycolaldehyde. With a further increase in the potential, a distinct peak at 1309 cm^–1^, corresponding to the COO^–^ vibration of oxalic acid, appeared, implying that GA underwent further oxidation. Finally, at potentials above 1.15 V_RHE_, the signal associated with oxalic acid diminished and disappeared, while the emergence of a peak at 1638 cm^–1^ confirmed the formation of HCOOH, indicating the continued oxidation of GA accompanied by C–C bond cleavage.

### The Impact of Mn, Fe, and Co-Dopants on the Performance of Ni-Based Catalysts

In the previous sections, we have described the effect of different heteroatoms, especially 3d transition metals, on the OER and OOR activities of Ni­(O)­OH.^25,30,32^ From the above discussions, it is clear that Fe enforces a significant effect to improve the OER activity of Ni­(O)­OH, while for the organic oxidation reaction, Co and Mn play a specific role. In some cases, the second metal ions directly participate in the redox process required for the anodic oxidation, while the passive role of the metal ions in the redox process has also been reported. Although the second metal ion in Ni­(O)­OH can be active or passive in the redox process, its effect on the modulation of the electronic and local structure of the active catalyst is evident from different spectroscopic studies.^25,30,32^

Among the different dopants, Fe is particularly effective in enhancing the OER activity of Ni­(O)­OH by facilitating charge transfer and producing new active sites.^21,110–114^ However, the presence of excess Fe in the catalyst structure can lead to the separation of the Fe­(O)­OH phase, reducing the overall activity and stability.^21,110^ Further, Fe-rich phases suffer from higher dissolution of Fe compared to Ni or Co analogues, leading to a compromise between activity and long-term durability.^17,18,20,39^ Selective leaching and redeposition of Fe from Fe–Ni­(O)­OH can help maintain high activity through dynamic surface reconstruction.

To understand the effect of Fe incorporation in Ni­(O)­OH to improve the OER activity, operando and in situ spectroscopic studies, along with DFT calculations, were conducted. The combination of these studies revealed the following points:

Ni as the lone active center: A large number of FeNi­(O)­OH catalysts have been reported for the OER, in which Ni serves as the primary active site, while Fe acts as a promoter, enhancing the density of electrochemically active sites, improving electronic conductivity, and optimizing the reaction kinetics.^18,20,30^

Ni, Fe dual active center: Beyond single-active-site systems, a few reports showed the cooperative role of both Ni and Fe active sites during OER.^21,110–114^

However, Fe–Ni­(O)­OH is a poor choice for OOR compared to OER because OER is a redox-active process, while OOR primarily follows a diffusion-controlled route. The addition of iron to Ni catalysts produces three key detrimental effects: (i) it decreases current density; (ii) it shifts the redox peak anodically; and (iii) it shifts the OOR onset to a higher potential.

Furthermore, from the chosen metals, Co-based catalysts are particularly attractive for both OER and OOR due to their flexible oxidation states, favorable reversibility, outstanding thermal and mechanical stability, and cost-effectiveness.^101,102^ Our group and others have observed that the addition of the Co^2+/3+^ ion increases the conductivity of the active catalyst, facilitating the facile oxidation of Ni^2+^ to Ni^3+^(O)­OH, the main active catalyst in organic oxidation reactions.^101,102^ For example, in the case of PhCH_2_OH oxidation, Ni­(O)­OH shows poor activity, but the introduction of Co facilitates Ni^2+/3+^ oxidation and thereby increases overall activity. Similar results were also observed with UOR by others.^100–102^

In situ XAS studies have revealed that Co^3+^ and Fe^3+^ can substitute the Ni within the NiO_6_ octahedra without inducing significant lattice distortion. The Fe- and Co-containing octahedra act as adaptive structural units that accommodate the expansion of the Ni­(O)­OH lattice while mostly preserving the Fe–O and Co–O bond distances. In contrast, Mn incorporation is more complex. Mn^3+^ is more prone to cation exchange than Mn^2+^ because its ionic radius is better suited for octahedral coordination.^113^ However, unlike the electron-withdrawing effects of Co^3+^ and Fe^3+^, Mn^3+^ possesses a relatively strong reducing character and therefore is expected to lower the average oxidation state of Ni^3+^. Although Mn^4+^ can improve structural strain by suppressing Jahn–Teller distortions, its smaller ionic radius and weaker preference for octahedral coordination increase its tendency to migrate toward tetrahedral sites.^113–115^

In contrast, the electronegativity difference between Mn (1.55) and Ni (1.91) promotes charge redistribution within the lattice structure, thereby enhancing catalytic activity toward the OOR.^115^ Moreover, the incorporation of Mn^3+^ induces Jahn–Teller distortion due to its electronic configuration in an octahedral crystal field.^115^ This distortion results in anisotropic variations in bond lengths within the lattice, which consequently modulate the catalyst’s electrical conductivity, redox behavior, electronic structure, and overall catalytic performance.^113–115^

Consequently, Mn-doped Ni catalysts provide a strong framework that facilitates effective charge transfer and ion diffusion. Moreover, tuning the Mn/Ni ratio significantly influences the oxidation states, reducibility, and overall catalytic performance.^116,117^ Furthermore, the defect-rich sites in Mn–Ni­(O)­OH provide greater availability of the deprotonated sites, thereby enhancing the covalency of the Ni–O bonds and weakening the O–H bonds. As a result, NiMn-based catalysts are more suitable for OORs than OER.^116,117^ In a related study, Ding et al. examined 3d metal-doped α-Ni­(OH)_2_ for amine oxidation and found that Mn doping enhanced amine adsorption on γ-NiOOH while preserving oxygenated species adsorption, delivering the highest activity and selectivity among the tested mono- and bimetallic catalysts.^72^

Based on this observation, it can be concluded that the second metal ions with higher oxidation states (M^3+δ/4+^) are advantageous for enhancing OER performance, whereas those with lower oxidation states (M^2+δ/3+^) are useful for organic oxidation reactions. Additionally, the improved electronic conductivity in Co-containing Ni­(O)­OH can be attributed to the introduction of holes into the partially filled, low-spin Co^3+/4+^: t_2g_^6–*x*^ band, as well as direct metal-to-metal interactions across the shared octahedral edges within the slab. In contrast, the evenly filled bands in Mn^4+^ result in semiconducting behavior.

Therefore, with a Ni-based catalyst, the OOR takes place at a sufficiently high potential, thereby facilitating the accumulation of highly valent Ni^3+^ within the active catalyst. These states promptly drive HAT, or hydride transfer, from the organic reactant. Furthermore, it is possible that electrophilic active oxygen species originating at Ni^3+/4+^(O)­OH expedite the proton abstraction from organic substrates. Regardless, the outcome is the formation of Ni^2+^-oxo species. This phenomenon holds true for alcohols, amines, HMF, plastics, and aldehyde oxidation studied so far, irrespective of the structural differences in the Ni-based catalysts.

### Exception: Catalysts beyond Ni^3+^(O)­OH for OOR

Mostly, the reported Ni-based catalysts are in situ activated into Ni­(O)­OH via surface reconstruction during the OOR.^25,110–114^ The Ni^3+^ species in the active catalyst provide the active sites for the OOR. However, some recent reports have shown the active participation of Ni^2+^ species in the OOR process instead of Ni^3+^. For example, Geng et al. reported Ni_2_[Fe­(CN)_6_] for UOR with Ni^2+^ active sites.^96^ Although most of the reports have explained Ni^3+^ as the active site for UOR, the main limitation arises from the strong binding affinity of Ni^3+^ toward the *COO intermediate.^101,102^ This excessively strong adsorption disfavors intermediate desorption, disrupts the energy balance of the reaction process, and ultimately results in sluggish kinetics and reduced activity.^118^

In contrast, Ni^2+^ exhibits comparatively weaker *COO adsorption, facilitating intermediate desorption and regeneration of more accessible active sites, and promoting faster electron transfer to enhance the overall reaction rate.^118^ Moreover, the activation energy for the reactions catalyzed on Ni^2+^ sites is lower than that on Ni^3+^.

Furthermore, Yang groups reported a unique Ni^2+^ site in NiFe single-crystalline Prussian blue analogue (NiFe-sc-PBA) that acts as a stable, efficient, and selective active center for EG electrooxidation toward formic acid.^119^ In situ and operando characterizations confirmed the structural robustness of the Ni^2+^ sites during EG electrooxidation. Furthermore, molecular dynamics simulations revealed that EG molecules preferentially accumulate on the NiFe-sc-PBA surface, thereby suppressing hydroxyl-induced surface reconstruction under alkaline conditions.^119^ Including this example in our manuscript will help clarify that Ni­(O)­OH is not universally required for OOR activity, and that alternative active phases—such as cooperative multisite pathways within coordination networks—can bypass reconstruction. Furthermore, for the selective oxidation of HMF to FDCA, the Chen group reported Ni^2+^-stabilized catalyst featuring a Ni^2+^–O-Pd interfacial structure. The Ni^2+^–O-Pd interface decreases the C–H activation barrier and stabilizes the catalytic sites.^120^

## Conclusions and Perspective

In this perspective, we emphasize the following frontline aspects:

Efficient Catalyst System: Although substantial progress has been achieved in the Ni-based catalysts for different anodic oxidation reactions, a precise structure–activity–selectivity relationship is still missing. We have found the following parameters that can provide an idea about the design of an efficient catalyst system for OER and different OORs.
  Oxidation State Modulation: The studies on electrochemical OER and OOR suggest that the catalytic onset potential is predominantly determined by the electrocatalyst’s favorable oxidation state rather than its intrinsic thermodynamic properties.
  Dynamic Redox Behavior: A key feature of Ni-based electrocatalysts is the anodic activation to the active catalyst Ni­(OH). The dynamic redox behavior, enabling reversible transitions between oxidation states (Ni^2+^/Ni^3+^/Ni^4+^) under electrochemical conditions, is essential to achieve high activity. In this context, the heteroatom doping in Ni­(O)­OH plays a key role.
  Heteroatom Doping: Although the Fe incorporation in Ni­(O)­OH produces a high OER activity, it leads to a poorer OOR than the incorporation of Co or Mn. In most cases, Co also provides the active site for the OOR, thus improving the overall catalytic activity.^61^ In contrast, Mn in Mn–Ni­(O)­OH does not offer the active sites for the binding of the substrates; rather, it modulates the electronic structure of the active catalyst to produce a better OOR activity.^30,64,65^

    Although Fe^3+^ significantly enhances the OER activity of Ni­(O)­OH, it complicates mechanistic analysis and the study of the intrinsic redox behavior of Ni. While the reported operando spectroscopic studies provide important information about the dynamic structural changes of the catalyst at the anode, in situ characterization of the anodized catalyst surface and its bulk structure would provide deeper insight into the mechanisms responsible for the enhanced activity.
Modulation of Reaction Mechanism: Interestingly, the doping of heteroatoms, defect generation, strain and phase modulation, oxygen vacancy concentration, lattice oxygen mobility, defect density, etc., in Ni­(O)­OH changes the reaction mechanism of OER and OOR. However, the mechanistic study is mostly focused on the theoretical calculations, which consider only limited parameters instead of explaining the overall features of the catalyst structure. Therefore, future research should combine spectroscopic studies with the theoretical calculations for a better understanding of the reaction mechanism.
  Although XAS and Raman spectroscopies have emerged as powerful tools for the precise tracking of the elemental and local structural environments of Ni-based catalysts, they cannot fully distinguish catalysts with similar electronic structures but contrasting activities, thereby limiting mechanistic insight. Future oxygen K-edge XAS and Resonant Inelastic X-ray Scattering (RIXS) can be utilized to probe the metal–oxygen covalency, charge transfer, and detect the active intermediates. Such efforts are crucial for understanding dopant effects and guiding the rational design of efficient Ni-based OER and OOR catalysts.
Performance Evaluation of the Catalysts: The Ni-based OOR catalysts also show high OER activity. As a result, the competition between OER and OOR in the aqueous medium reduces the FE of OOR. In general, the formation of Ni^IV^=O species favors the O–O bond formation, while Ni^3+^ participates in OOR. Therefore, a substantial potential gap between the Ni^2+/3+^ and Ni^3+/4+^ oxidation peaks can offer a window for OOR to achieve high FE. Further, increasing the hydrophobicity of the electrode surface, pH control, and the use of different electrolytes can offer better FE for OOR.
  A direct comparison of the different Ni-based catalysts for a particular organic oxidation/OER is almost impossible, as the reaction conditions are varied in different studies. Similarly, finding a suitable substrate to replace the OER at the anode is difficult, as the catalyst is massively varied in the different reports. To address this point, we and a few other groups have studied the anodic oxidation of a series of organic compounds employing a single catalyst to identify a proper OOR as a replacement for OER.^25,30,96^

Scaling-Up Reaction and Product Separation: Most of the reported studies deal with a very low concentration of organic substrates (less than 10 mM), and for real applications, a high concentration of the substrate is necessary. At the same time, the separation of products like benzoic acid from the electrolyte requires the neutralization of the alkaline solution with a huge amount of acid; this is definitely not suitable for practical applications. Therefore, catalyst design for a neutral or near-neutral medium is essential.^76,77^ However, some recent reports pave the route to success with a high concentration of substrates and conducting OOR in a near-neutral medium with high activity and product selectivity.^121^
Emerging OER Regulation Strategies: The OER is kinetically sluggish, and anodic reconstruction typically requires a high potential (≥1.40 V vs RHE) to generate the active catalyst. To lower the reconstruction potential, accelerate kinetics, and improve energy efficiency, several emerging strategies can be applied:^122–126^
  (Photothermal Effect: Localized heat generated under light irradiation increases molecular collision frequency and reduces kinetic barriers, promoting faster reconstruction.
  Thermal Heating: Elevated temperature enhances Brownian motion, accelerating the diffusion of reactants and intermediates, and thus, reconstruction kinetics.
  Magnetothermal Heating: Alternating magnetic fields induce local hyperthermia in magnetic nanoparticles, generating intense on-site heat that speeds up the reconstruction.
  Solar-Driven Thermal Annealing: Focused solar irradiation enables rapid structural transformation and crystallization, offering a fast alternative to conventional heating.
  Spin Polarization: Electronic structure modulation and chirality-induced spin polarization of the active center can also improve the electrochemical OER/OOR activity of the Ni-based catalysts.
Potential Applications: A careful analysis of the Ni-based catalysts for the anodic oxidation reactions has led us to the conclusion that, although they show promising activities, there is a lot of unrealized potential that could spur more research and development. In addition to the spectroscopic and theoretical studies, the utilization of artificial intelligence and deep machine learning could be ideal for designing efficient Ni-based catalyst systems.
